# Novel Biomarkers in Patients with Chronic Kidney Disease: An Analysis of Patients Enrolled in the GCKD-Study

**DOI:** 10.3390/jcm9030886

**Published:** 2020-03-24

**Authors:** Moritz Mirna, Albert Topf, Bernhard Wernly, Richard Rezar, Vera Paar, Christian Jung, Hermann Salmhofer, Kristen Kopp, Uta C. Hoppe, P. Christian Schulze, Daniel Kretzschmar, Markus P. Schneider, Ulla T. Schultheiss, Claudia Sommerer, Katharina Paul, Gunter Wolf, Michael Lichtenauer, Martin Busch

**Affiliations:** 1 Department of Internal Medicine II, Division of Cardiology, Paracelsus Medical University of Salzburg, 5020 Salzburg, Austria; m.mirna@salk.at (M.M.); a.topf@salk.at (A.T.); b.wernly@salk.at (B.W.); r.rezar@salk.at (R.R.); v.paar@salk.at (V.P.); k.kopp@salk.at (K.K.); u.hoppe@salk.at (U.C.H.); 2 Department of Cardiology, Pulmonology and Vascular Medicine, Medical Faculty, Heinrich Heine University Duesseldorf, 40225 Duesseldorf, Germany; christian.jung@med.uni-duesseldorf.de; 3 Department of Internal Medicine I, Division of Nephrology, Paracelsus Medical University of Salzburg, 5020 Salzburg, Austria; h.salmhofer@salk.at; 4 Department of Internal Medicine I, Division of Cardiology, Friedrich Schiller University Jena, 07743 Jena, Germany; christian.schulze@med.uni-jena.de (P.C.S.); daniel.kretzschmar@med.uni-jena.de (D.K.); 5 Department of Nephrology and Hypertension, University Hospital Erlangen, Friedrich-Alexander University Erlangen-Nürnberg, 91054 Erlangen, Germany; markus.schneider@klinikum-nuernberg.de; 6 Department of Medicine IV – Nephrology and Primary Care, Institute of Genetic Epidemiology, Medical Center–University of Freiburg, Faculty of Medicine, 79106 Freiburg, Germany; ulla.schultheiss@uniklinik-freiburg.de; 7 Department of Nephrology, University of Heidelberg, 69117 Heidelberg, Germany; claudia.sommerer@med.uni-heidelberg.de; 8 Department of Internal Medicine III, Friedrich Schiller University Jena, 07743 Jena, Germany; katharina.paul@med.uni-jena.de (K.P.); gunter.wolf@med.uni-jena.de (G.W.); martin.busch@med.uni-jena.de (M.B.)

**Keywords:** CKD, CVD, biomarkers, sST2

## Abstract

*Background:* Chronic kidney disease (CKD) and cardiovascular diseases (CVD) often occur concomitantly, and CKD is a major risk factor for cardiovascular mortality. Since some of the most commonly used biomarkers in CVD are permanently elevated in patients with CKD, novel biomarkers are warranted for clinical practice. *Methods:* Plasma concentrations of five cardiovascular biomarkers (soluble suppression of tumorigenicity (sST2), growth differentiation factor 15 (GDF-15), heart-type fatty acid-binding protein (H-FABP), insulin-like growth factor-binding protein 2 (IGF-BP2), and soluble urokinase plasminogen activator receptor) were analyzed by means of enzyme-linked immunosorbent assay (ELISA) in 219 patients with CKD enrolled in the German Chronic Kidney Disease (GCKD) study. *Results:* Except for sST2, all of the investigated biomarkers were significantly elevated in patients with CKD (2.0- to 4.4-fold increase in advanced CKD (estimated glomerular filtration rate (eGFR) < 30 mL/min/1.73 m² body surface area (BSA)) and showed a significant inverse correlation with eGFR. Moreover, all but H-FABP and sST2 were additionally elevated in patients with micro- and macro-albuminuria. *Conclusions:* Based on our findings, sST2 appears to be the biomarker whose diagnostic performance is least affected by decreased renal function, thus suggesting potential viability in the management of patients with CVD and concomitant CKD. The predictive potential of sST2 remains to be proven in endpoint studies.

## 1. Introduction

Chronic kidney disease (CKD) affects about 11.5% of the overall population with increasing age-dependent prevalence of up to 47% in persons older than 70 years [1]. Apart from old age, CKD is associated with diabetes mellitus and hypertension. Due to an increase of these precipitating and often causative diseases, the prevalence of CKD is expected to rise even further in the future [1]. Because of shared risk factors and the fact that CKD constitutes an independent risk factor itself, CKD and cardiovascular disease (CVD) often occur concomitantly [2,3,4]. Hence, biomarkers established in the evaluation of patients with CVD are increasingly used in patients with decreased renal function. Unfortunately, some of the most common biomarkers in this field, such as troponin or brain natriuretic peptide (BNP), are chronically elevated in patients with CKD, which may in part be due to impaired renal clearance [5,6,7]. Therefore, their clinical applicability in patients with CKD is limited and hence, novel biomarkers are warranted to improve diagnosis and risk stratification in these disease entities.

In the following study, plasma concentrations of novel cardiovascular biomarkers (sST2, GDF-15, H-FABP, IGF-BP2 and suPAR) were investigated in patients with various stages of CKD.

Soluble suppression of tumorigenicity (sST2; molecular mass: 36,993 Da [8]; normal reference ranges for male subjects: 4000–31,000 pg/mL; for female subjects: 2000–21,000 pg/mL [9]) is a member of the toll-like/IL-1-receptor family that acts as a scavenger-receptor for IL-33, thus attenuating the effects of this immunomodulatory cytokine [10]. sST2 is secreted in response to mechanical stress, and hence elevated plasma levels are found in patients with acute and chronic heart failure [11]. Increased plasma concentrations of sST2 have been associated with adverse outcomes in patients with coronary artery disease [12] and heart failure [11,13] in previous trials.

Growth differentiation factor 15 (GDF-15; molecular mass: 34,140 Da [14]; normal reference ranges: 310 ± 10 pg/mL [15]) is a member of the transforming growth factor ß (TGF-ß) cytokine family. GDF-15 is secreted in response to tissue injury or by the effect of proinflammatory cytokines and is involved in the regulation of inflammatory and apoptotic processes [16]. Recently, elevated plasma levels of GDF-15 have been associated with an increased risk of mortality in patients with coronary artery disease and chronic heart failure [17,18,19]. Furthermore, increased plasma concentrations of circulating GDF-15 were associated with a decline of renal function in patients with CKD [20].

Heart-type fatty acid-binding protein (H-FABP; molecular mass: 14,858 Da [21]; normal reference ranges for male subjects: 3.5 ± 0.4; for female subjects: 3.9 ± 0.4 ng/mL [22]) is a small cytoplasmic protein that transports long-chained fatty acids in cardiomyocytes and is considered a biomarker of myocardial ischemia [23]. In case of damage to the cell membrane, H-FABP is rapidly released into circulation and therefore was evaluated for use in diagnosis and risk stratification of coronary artery disease and acute coronary syndrome [24,25]. In fact, increased plasma levels of H-FABP are associated with an elevated risk of adverse outcomes in acute coronary syndrome and heart failure [26,27].

Insulin-like growth factor-binding protein 2 (IGF-BP2; molecular mass: 34,814 Da [28]; normal reference ranges: 321.2 ± 285.0 ng/mL [29]) is an anabolic peptide with extensive structural and functional homology to insulin. IGF-BP2 is a potent effector of growth, proliferation, and metabolism that elicits its effects via autocrine, paracrine, and endocrine mechanisms [30]. Elevated plasma concentrations of IGF-BP2 have been associated with diabetes mellitus [31], metabolic syndrome [32], and progression of CKD [33] in previous studies. Moreover, IGF-BP2 seems to be involved in the pathogenesis of atherosclerosis. In a recent trial, plasma concentrations of IGF-BP2 were inversely correlated with arterial intima-media thickness of the carotid artery in healthy participants [34,35].

Soluble urokinase plasminogen activator receptor (suPAR; molecular mass (depending on the considered isoform): 31,263–36,978 Da [36]; normal reference ranges: 2100 pg/mL, IQR: 1700–2300 pg/mL [37]) is the soluble isoform of the urokinase plasminogen activator receptor (uPAR), a membrane-bound protein in endothelial and immunological cells that plays a role in various inflammatory processes [38]. Recent evidence suggests that suPAR is involved in the formation of atherosclerotic lesions and hence, elevated plasma levels of suPAR have been associated with an increased risk for coronary artery disease and cardiovascular mortality [39]. Furthermore, elevated plasma concentrations of suPAR were recently correlated with the deterioration of renal function in patients with CKD [40,41], and an association between suPAR and primary focal segmental glomerulosclerosis (pFSGS) [42,43] was found.

## 2. Materials and Methods

Plasma samples from 219 of 245 patients enrolled in the regional center of Jena within the German Chronic Kidney Disease study (GCKD), Germany, were analyzed. The remaining 26 patients were excluded as serum samples were missing. The GCKD study was approved by the local ethics committee, registered in the German national registry for clinical studies (DRKS00003971) and was conducted according to the principles of the Declaration of Helsinki and Good Clinical Practice. Informed consent was obtained from all patients prior to enrollment.

### 2.1. Study Population

Details of the study design and the enrollment process of the GCKD study have been described previously [44]. Briefly, patients aged 18–74 years with CKD in routine nephrological care were enrolled across nine German study centers between March 2010 and March 2012. Patients were included if they had an estimated glomerular filtration rate (eGFR) of < 60mL/min/1.73m² body surface area (BSA) or overt proteinuria in the presence of a higher eGFR (defined as albuminuria of > 300 mg/g creatinine or proteinuria of > 500 g/g creatinine). Exclusion criteria were non-Caucasian race, history of transplantation, active malignancy, New York Heart Association (NYHA) heart failure functional class IV, and/or inability to provide written informed consent [44].

Glomerular filtration rate (GFR) was estimated using the 4-variable modification of diet in renal disease (MDRD) formula, as previously published [45,46]. CKD was categorized according to the clinical practice guidelines from the Kidney Disease: Improving Global Outcomes Initiative (KDIGO) in the following G and A-stages. G-stages: CKD stage G1: eGFR ≥ 90 mL/min/1.73 m² BSA, stage G2: eGFR 60–89 mL/min/1.73 m² BSA, stage G3a: 45–59 mL/min/1.73 m² BSA, stage G3b: eGFR 30–44 mL/min/1.73 m² BSA, and stages G4 and G5 (combined): eGFR < 30 mL/min/1.73 m² BSA. A-stages: urinary albumin/creatinine ratio (UACR) < 30 mg/g Crea (A1 = normo-albuminuria), 30–300 mg/g Crea (A2 = micro-albuminuria), or > 300 mg/g Crea (A3 = macro-albuminuria) [47,48]. Symptoms of heart failure were estimated by the modified Gothenburg scale, as previously published [47,49].

### 2.2. Blood Samples and Biomarker Analysis

Blood samples were collected upon study enrollment using a vacuum-containing system. Plasma levels of sST2, GDF-15, H-FABP, IGF-BP2, and suPAR were measured by using commercially available enzyme-linked immunosorbent assay (ELISA) kits (R&D Systems, USA). Preparation of reagents and measurements were performed according to the manufacturer’s instructions. In brief, patient samples and standard protein were added to the wells of the ELISA plates (Nunc MaxiSorp flat-bottom 96 well plates, VWR International GmbH, Austria) and incubated for two hours. Plates were then washed using a Tween 20/PBS solution (Sigma Aldrich, USA). Then, a biotin-labelled antibody was added and incubated for another two hours. Plates were washed another time, and streptavidin–horseradish-peroxidase solution was added to the wells. After adding tetramethylbenzidine (TMB; Sigma Aldrich, USA) a color reaction was generated. Values of optical density (OD) were determined at 450 nm on an ELISA plate-reader (iMark Microplate Absorbance Reader, Bio-Rad Laboratories, Austria).

### 2.3. Statistical Analysis

Statistical analyses were performed using SPSS (Version 24.0, SPSSS Inc., USA) and GraphPad Prism software (GraphPad Software, USA). Normally distributed data was expressed as mean and standard deviation (SD); not normally distributed data was expressed as median and interquartile range (IQR). Medians were compared using a Mann–Whitney U-test or a Kruskal–Wallis test with Dunn’s post-hoc test, depending on the number of groups analyzed. Bonferroni–Holm correction was conducted to adjust for multiple comparisons. To assess the association between renal function and biomarker concentrations, correlation analysis was conducted using Spearman’s rank correlation test, followed by multiple linear regression analysis to adjust for parameters known for confounding with renal function (age, gender, BMI, diabetes mellitus, and arterial hypertension). Prior to multiple linear regression analysis, normal distribution was assessed by performing a Kolmogorov–Smirnov test, where applicable, and multicollinearity was excluded using the collinearity diagnostics tool by SPSS. A *p*-value < 0.05 was considered statistically significant.

## 3. Results

In total, 219 plasma samples of patients enrolled in the GCKD study were analyzed. The mean age was 63 ± 9 years, and the majority of patients were male (60.3%, n = 132). Regarding comorbidities, arterial hypertension was present in 90.4% (n = 198), diabetes mellitus type 2 in 39.3% (n = 86), heart failure in 26.0% (n = 57), and 49.3% had a history of smoking (n = 108) (see Table 1).

### 3.1. Renal Function and Causes of Renal Disease

Regarding renal function, the majority of patients was in CKD stages G3a (41.6% (n = 91), eGFR 45–59 mL/min/1.73 m² BSA) and G3b (32.4% (n = 71), eGFR 30–44 mL/min/1.73 m² BSA), followed by CKD stage 2 (13.7% (n = 30), eGFR 60–89 mL/min/1.73 m² BSA) and CKD stages 4 and 5 (9.6% (n = 21), eGFR < 30 mL/min/1.73 m² BSA); 2.7% (n = 6) of the patients had an eGFR above 90 mL/min/1.73 m² BSA while having proteinuria.

Regarding urinary albumin excretion, micro-albuminuria (UACR 30–300 mg/g) was observed in 32% (n = 70) of patients, whereas macro-albuminuria (UACR > 300 mg/g) was evident in 20.5% (n = 45) of the patients at the time of inclusion (see Table 1). Only two patients had an UACR above 3000 mg/g.

The median estimated glomerular filtration rate (eGFR) was 47.7 mL/min/1.73 m² (IQR 38.2–55.7), the median level of creatinine was 1.5 mg/dL (IQR 1.2–1.7), and the median level of cystatin-C was 1.4 mg/L (IQR 1.2–1.7). The median plasma level of serum urea was 26.5 mg/dL (IQR 20.6–33.3), the median level of uric acid was 7.1 mg/dL (IQR 6.0–8.3), and the median level of CRP was 2.4 mg/dL (IQR 1.2–4.9).

The leading cause of renal disease was nephrosclerosis (28.8%, n = 63), followed by diabetic nephropathy (diabetes mellitus type 1 and 2 combined: 17.4%, n = 38) and interstitial nephropathy (9.1%, n = 20) (see Table 1).

### 3.2. Biomarker Concentrations

**Figure 1:**
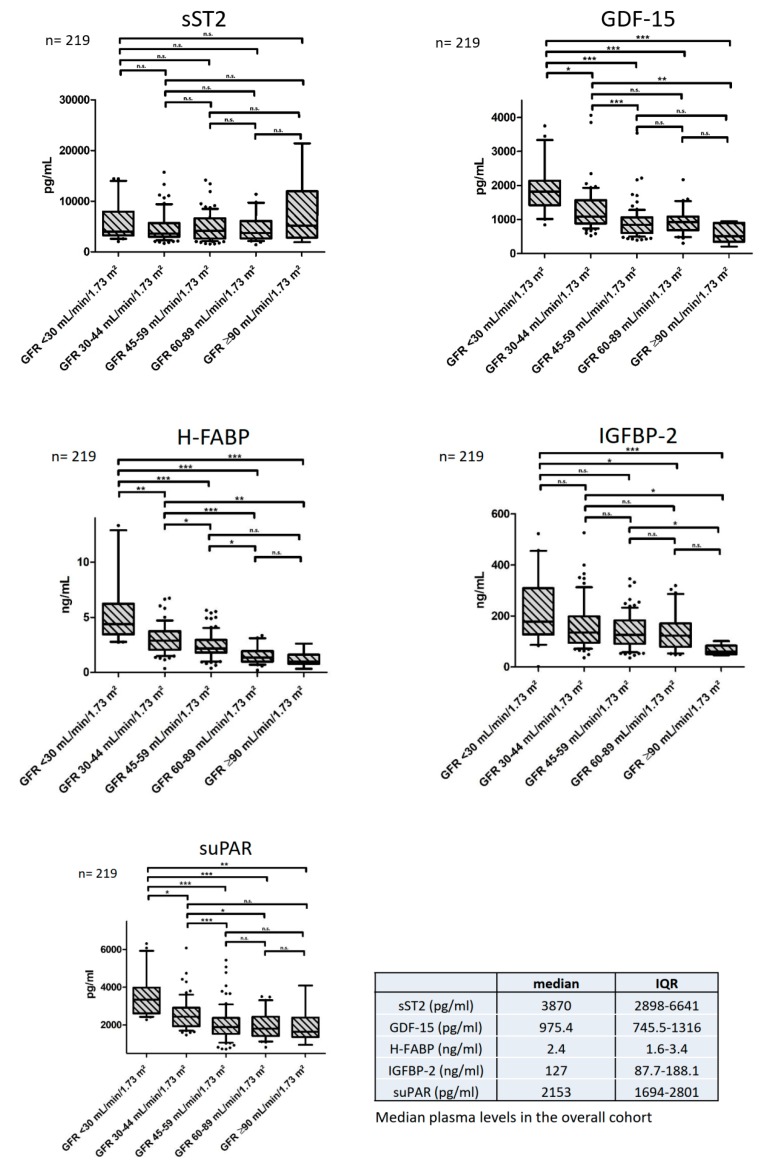
Biomarker concentrations throughout the stages of CKD. Median plasma levels and interquartile ranges (IQR) of the overall cohort are depicted in the additional table. * indicates a *p* of <0.05, ** a *p* of <0.01 and *** a *p* of <0.001, n.s.= not significant. Abbreviations: sST2 = soluble suppression of tumorigenicity, GDF-15 = growth differentiation factor 15, H-FABP = heart-type fatty acid binding protein, IGF-BP2= insulin-like growth factor binding protein 2, suPAR = soluble urokinase plasminogen activator receptor, eGFR = estimated glomerular filtration rate, IQR = interquartile range.

The median plasma levels of sST2, GDF-15, H-FABP, IGF-BP2, and suPAR in our study cohort are depicted in Figure 1 and Supplementary Materials, Table A1 in Appendix A.

Except for sST2, all of the investigated biomarkers showed significantly elevated plasma concentrations in the advanced stages of CKD (GDF-15: 3.6-fold increase, H-FABP: 4.4-fold increase, IGF-BP2: 3.0-fold increase, suPAR 2.0-fold increase when eGFR was <30 mL/min/1.73 m² BSA compared to eGFR ≥ 90 mL/min/1.73 m² BSA, see Figure 1 and Supplementary Materials, Table A1 in Appendix A). This finding remained statistically significant after applying Bonferroni–Holm correction for multiple comparisons (GDF-15: *p* = 0.0005, H-FABP: *p* = 0.0005, IGF-BP2: *p* = 0.002, suPAR: *p* = 0.0005).

Patients with concomitant symptoms of heart failure had significantly elevated plasma concentrations of sST2 (median 5039 pg/mL vs. 3673 pg/mL, *p* =0.008).

### 3.3. Correlation Analyses and Multiple Linear Regression Analyses

**Figure 2:**
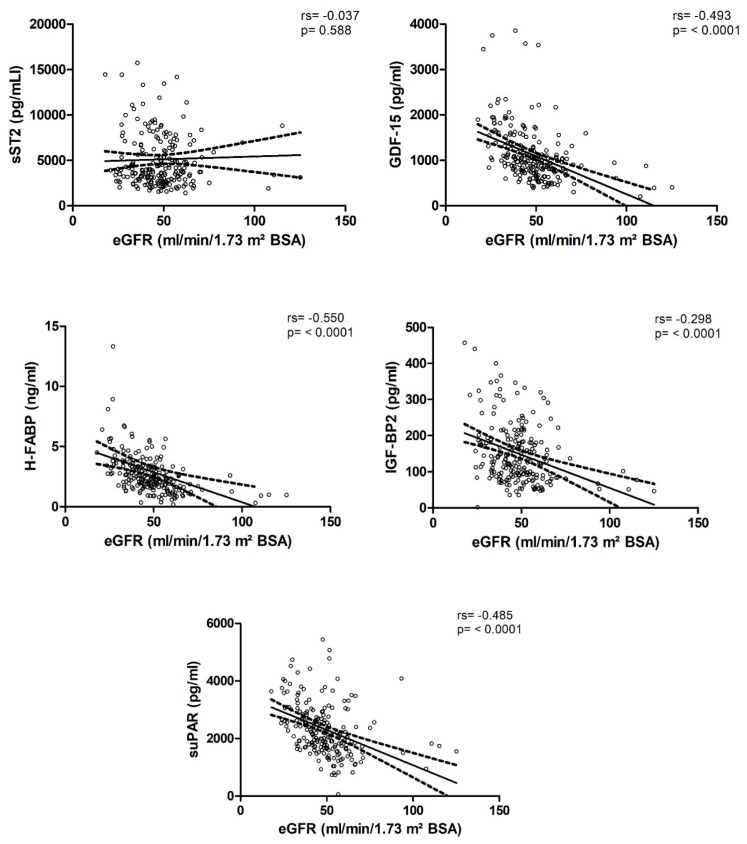
Visual representation of the correlation of biomarker concentrations with estimated glomerular filtration rate (eGFR).

Plasma concentrations of GDF-15, H-FABP, suPAR, and IGF-BP2 showed a significant positive correlation with serum creatinine (GDF-15: rs = 0.566, *p* < 0.0001, H-FABP: rs = 0.584, *p* < 0.0001, suPAR: rs = 0.506, *p* < 0.0001, IGF-BP2: rs = 0.267, *p* < 0.0001; rs = correlation coefficient) and a significant inverse correlation with eGFR (GDF-15: rs = −0.493, *p* < 0.0001, H-FABP: rs = −0.550, *p* < 0.0001, suPAR: rs = −0.485, *p* < 0.0001, IGF-BP2: rs = −0.298, *p* < 0.0001), which remained statistically significant after applying Bonferroni–Holm correction. sST2 showed no correlation with renal function, neither with serum creatinine, nor with eGFR (see Figure 2 and Table 2).

The correlation of biomarker concentrations with eGFR remained statistically significant in a multiple linear regression analysis after correction for parameters that are known to confound with renal function (GDF-15: B = −0.10, *p* < 0.0001; H-FABP: B = −1.187, *p* < 0.0001; IGF-BP2: B = −0.064, *p* < 0.0001; suPAR: B = −0.006, *p* < 0.0001; B = regression coefficient, see Supplementary Materials, Table A2 in Appendix A). There was still no significant correlation between plasma concentrations of sST2 and renal function (sST2: B = 0.000, 95% CI 0.000–0.001, *p* = 0.643) after adjusting for the aforementioned confounders.

Except for H-FABP, all of the biomarkers showed a weak, yet statistically significant correlation with the UACR (sST2: rs = 0.139, *p* = 0.044; GDF-15: rs = 0.251, *p* < 0.0001; IGF-BP2 rs = 0.192, *p* = 0.005; suPAR: rs = 0.163, *p* = 0.018, H-FABP: rs = 0.100, *p* = 0.149, see Table 2). Notably, the weak correlations of sST2 and suPAR with the UACR were statistically insignificant after applying the Bonferroni–Holm correction for multiple comparisons (sST2: *p* = 0.088, suPAR: *p* = 0.054).

However, after adjusting for the aforementioned confounders in another multiple linear regression model, all correlations with the UACR, except the ones with suPAR and H-FABP, remained statistically significant (sST2: B = 0.031, *p* = 0.007; GDF-15: B = 0.179, *p* = 0.012; IGF-BP2: B = 2.086, *p* < 0.0001; see Supplementary Materials, Table A2 in Appendix A).

Furthermore, the plasma concentrations of suPAR, H-FABP, and IGF-BP2 showed a significant correlation with BMI and the plasma levels of suPAR, H-FABP, and GDF-15 correlated with CRP. Additionally, the plasma concentrations of H-FABP correlated with the plasma levels of sST2, GDF-15, suPAR, and IGF-BP2, and the concentrations of suPAR correlated with the plasma levels of GDF-15 and IGF-BP2 and vice versa (see Table 2).

### 3.4. Biomarker Concentrations in Patients with Albuminuria

The plasma levels of GDF-15, IGF-BP2, and suPAR were significantly elevated in patients with micro-and macro-albuminuria, as defined by the UACR (Table 3). In contrast, the plasma concentrations of sST2 and H-FABP were not significantly influenced by the stage of albuminuria (see Table 3). This finding remained statistically significant after applying a Bonferroni–Holm correction for multiple comparisons (GDF-15: *p* = 0.01, IGF-BP2: *p* = 0.01, suPAR: *p* = 0.012).

## 4. Discussion

In patients with chronic kidney disease (CKD), a high burden of cardiovascular diseases (CVD) is common, and an inverse correlation of renal function with the prevalence of coronary artery disease, congestive heart failure, and cerebrovascular disease is observed [1,50]. Moreover, the incidence of acute kidney injury has been steadily increasing in recent years, leading to higher healthcare costs and mortality and contributing to increasing prevalence rates of CKD [51,52]. With an increasing prevalence of CKD from variable causes, the number of patients with end-stage renal disease is on the rise [1,53]. Furthermore, the presence of CKD markedly increases cardiovascular mortality in a stage-dependent manner [54,55,56]. According to current evidence, patients with end-stage renal disease (ESRD) undergoing hemodialysis have a 10- to 30-fold higher risk of cardiovascular mortality than the general population [57].

In fact, diagnosis, risk stratification, and treatment of patients with CVD increasingly relies on cardiovascular biomarkers. Since some of the most commonly used biomarkers for these purposes (e.g., troponin or brain natriuretic peptide (BNP)) are chronically elevated in patients with CKD [57,58], novel cardiovascular biomarkers are warranted to facilitate the management of patients with decreased renal function.

In our study cohort, plasma concentrations of GDF-15, H-FABP, IGF-BP2, and suPAR were markedly elevated in patients with decreased renal function, with a 2.0- to 4.4-fold increase in biomarker levels in the advanced stages of CKD (eGFR < 30 mL/min/1.73 m² BSA). In contrast, we found no significant elevation of sST2 in patients with CKD. In fact, the plasma levels of sST2 even remained unaltered in advanced CKD (eGFR < 30 mL/min/1.73 m² BSA) and showed no correlation with estimated glomerular filtration rate (eGFR). In contrast to sST2, we found significant correlations of the plasma levels of GDF-15, H-FABP, IGF-BP2, and suPAR with serum creatinine and eGFR. Considering potential diagnostic value, it is essential to determine whether a biomarker would accumulate due to impaired renal clearance or increase due to the pathophysiologic process that it is supposed to portray (i.e., troponin in myocardial ischemia). Although the association with renal function does not preclude the predictive ability of a biomarker, its clinical applicability in the evaluation of patients with CVD and concomitant CKD appears to be somewhat limited [59]. Since some of the most commonly used conventional biomarkers in CVD are chronically elevated in patients with CKD, at least partly because of impaired renal clearance, the finding that sST2 acts independently of renal function might be of significant relevance for clinical practice. Nevertheless, this finding needs to be confirmed in large prospective endpoint trials because it to some extent contradicts the findings of a study by Alam et al. In this study, some correlation of sST2 with renal function was observed in a larger, pooled cohort, yet this relationship was very weak [60]. Furthermore, the clinical performance of biomarkers needs to be confirmed in large prospective endpoint trials. In this regard, recent trials investigated the plasma concentrations of NT-proBNP, troponin T, and IGF-BP2 in patients with CKD and reported a higher prognostic value of the investigated biomarkers in these patients [61,62]. However, it is always questionable whether such studies consistently correct their statistical models for kidney function. Hence, the adjustment for renal function may be more valid for biomarkers, which do not primarily correlate with renal function. Interestingly, although the ST2/IL-33 signaling pathway seems to be involved in various inflammatory processes [63,64,65,66], the aforementioned study by Alam et al. did not find a statistically significant association of the plasma levels of sST2 with the progression of CKD to end-stage renal disease (ESRD) [60]. Taken together with our results, it seems as if sST2, in contrast to numerous other cytokines or mediators, acts relatively independent from renal function and pathophysiologic processes affecting the kidneys.

Furthermore, all of the investigated biomarkers, except for H-FABP and sST2, were additionally elevated in patients with micro- and macro-albuminuria, as defined by the UACR. This association is of particular interest, since albuminuria is an independent cardiovascular risk factor reflecting endothelial dysfunction [67,68], which might modulate the predictive potential of the biomarkers investigated. Notably, despite no statistical significance, we observed an obvious increase in the plasma concentrations of sST2 between the different stages of albuminuria (see Table 3). This increase was accompanied by a weak, yet statistically significant correlation of sST2 with albuminuria (sST2: rs = 0.139, *p* = 0.044), which became statistically insignificant after applying a Bonferroni–Holm correction for multiple comparisons.

sST2 is a promising new biomarker in risk stratification and therapy guidance [69,70] in patients with acute and chronic heart failure, and was associated with an increased risk of adverse outcomes in previous trials [12,71,72]. According to our present findings, sST2 might be a useful additional biomarker in the management of patients with CVD and concomitant CKD, with or without albuminuria. Although some studies reported similar findings in the plasma levels of sST2 in patients with CKD [73,74], the innovative value of our manuscript lies in the structured analysis and recording of five novel biomarkers, which portray different pathophysiological pathways, in a well-defined cohort. Furthermore, we investigated and described the respective plasma levels in different stages of CKD as reflected by eGFR and albuminuria.

## 5. Conclusions

Except for sST2, all of the investigated biomarkers were significantly elevated in patients with CKD, inversely correlating with eGFR. Based on our findings, sST2 appears to be the biomarker whose diagnostic performance is least affected by decreased renal function, hence suggesting potential viability in the management of patients with CVD and concomitant CKD. Whether this may influence its predictive potential in patients with CKD remains to be investigated by endpoint studies.

## 6. Limitations

A major limitation of this study is the absence of matched healthy controls, which would have further strengthened our findings. Moreover, the Gothenburg scale was found to be not ideal for reliably defining heart failure in patients with CKD in a previous trial due to shared symptoms and medications between the two disease entities [47]. However, a significant proportion of patients had concomitant heart failure, which may have acted as a bias in regard to the median concentrations of sST2. Notably, this trial did not analyze associations of the investigated biomarkers with clinical endpoints. We have to highlight that the conclusions drawn by the findings in this study are primarily of hypothesis-generating character and should be further validated in future trials. A limitation of the study may also be the applicability to populations of patients of non-Caucasian origin, since it is known that the cardiovascular risk also varies depending on ethnicity due to genetic differences. Thus, further investigative and population-specific endpoint trials, i.e., in the total GCKD cohort, seem necessary to confirm our present findings. One minor limitation is the use of estimated GFR instead of direct GFR measurement. Although more accurate, direct GFR measurement is too complex and impractical for everyday clinical use; hence, it appears unsuitable for a large multi-center trial. The use of eGFR by means of the MDRD formula does not represent a large bias regarding our current findings since only a minority of the patients had an eGFR above 60 mL/min/1.73 m².

## Figures and Tables

**Table 1:** Baseline characteristics, comorbidities, stages of chronic kidney disease (CKD), and causes of renal disease of the overall cohort.

**General**		
Age, mean (years)	63	±9
BMI, mean (kg/m²)	30	±5.6
Serum creatinine, median (mg/dl)	1.5	IQR 1.2–1.7
eGFR, median (mL/min/1.73 m²)	47.7	IQR 38.2–55.7
Urinary albumin/creatinine ratio (UACR), median (mg/g Crea)	44	IQR 7.4–216.7
**Comorbidities**	**%**	**(n)**
Hypertension	90.4	198
Diabetes mellitus	39.3	86
Heart Failure	26.0	57
**CKD stages**	**%**	**(n)**
Stage G1 (≥ 90 mL/min/1.73 m²)	2.7	6
Stage G2 (eGFR 60–89 mL/min/1.73 m²)	13.7	30
Stage G3a (eGFR 45–59 mL/min/1.73 m²)	41.6	91
Stage G3b (eGFR 30–44 mL/min/1.73 m²)	32.4	71
Stages G4 and G5 (eGFR <30 mL/min/1.73 m²)	9.6	21
**Urinary albumin/creatinine ratio (ACR)**	**%**	**(n)**
A1 (<30 mg/g)	43.8	96
A2 (30–300 mg/g)	32.0	70
A3 (>300 mg/g)	20.5	45
Missing	4.7	8
**Leading cause of renal disease**	**%**	**(n)**
Vascular nephrosclerosis	28.8	63
Diabetic nephropathy	17.4	38
Interstitial nephropathy	9.1	20
IgA-nephritis	4.1	9
Autosomal dominant polycystic kidney disease	4.1	9
Membranous glomerulonephritis	2.7	6
Membranoproliferative glomerulonephritis	1.4	3
Other	17.9	40
Missing	14.6	32

BMI = body mass index, DM = diabetes mellitus.

**Table 2:** Correlation analysis of the investigated biomarkers.

Biomarker		BMI	Creatinine	eGFR	UACR	CRP	sST2	GDF15	H-FABP	IGF-BP2	suPAR
sST2	rs	0.890	0.125	−0.037	0.139	0.087		0.133	0.348	0.151	0.082
	*p*-value	0.191	0.067	0.588	0.044 *	0.200		0.049	<0.0001	0.025	0.228
GDF-15	rs	0.097	0.566	−0.493	0.251	0.240	0.133		0.491	0.266	0.614
	*p*-value	0.151	<0.0001	<0.0001	<0.0001	0.0004	0.049		<0.0001	<0.0001	<0.0001
H-FABP	rs	0.314	0.584	−0.550	0.100	0.162	0.348	0.491		0.194	0.516
	*p*-value	<0.0001	<0.0001	< 0.0001	0.149	0.017	<0.0001	<0.0001		0.004	<0.0001
IGF-BP2	rs	-0.343	0.267	−0.298	0.192	−0.071	0.151	0.266	0.194		0.180
	*p*-value	<0.0001	<0.0001	<0.0001	0.005	0.296	0.025	<0.0001	0.004		0.007
suPAR	rs	0.243	0.506	−0.485	0.163	0.377	0.082	0.614	0.516	0.180	
	*p*-value	<0.0001	<0.0001	<0.0001	0.018 *	<0.0001	0.228	<0.0001	<0.0001	0.007	

* Denotes correlations that became statistically insignificant after applying a Bonferroni–Holm correction. Abbreviations: BMI = body mass index, CRP = C-reactive protein, eGFR = estimated glomerular filtration rate, rs = correlation coefficient, UACR = urinary albumin/ creatinine ratio.

**Table 3:** Concentrations in patients with normo-albuminuria, micro-albuminuria (UACR 30–300 mg/g, A2), and macro-albuminuria (UACR > 300 mg/g); the *p*-value represents the statistical differences between the three subgroups of albuminuria.

Biomarker	Total Cohort		Normo-albuminuria (A1)		Micro-albuminuria (A2)		Macro-albuminuria (A3)		
	median	IQR	median	IQR	median	IQR	median	IQR	*p*-Value
sST2 (pg/mL)	3870	2898–6641	3663	2726–6172	3647	2758–5793	4552	8587–3235	0.052
GDF-15 (pg/mL)	975.4	745.5–1316	892.2	675.6–1087	1035	780.9–861.7	1281	861.7–1635	0.002
H-FABP (ng/mL)	2.4	1.6–3.4	2.3	1.6–3.1	2.2	1.7–3.4	2.8	1.6–4.1	0.170
IGF-BP2 (ng/mL)	127	87.7–188.1	112.9	84.2–172.3	126.2	83.6–182.7	172.6	91.5–280.1	0.002
suPAR (pg/mL)	2153	1694–2801	1925	1653–2680	2197	1674–2723	2402	1918–2983	0.044

## Supplementary Materials

The following are available online at https://www.mdpi.com/2077-0383/9/3/886/s1, Table S1: Biomarker concentrations by estimated glomerular filtration rate (eGFR), Table S2: Multiple linear regression analysis with adjustment for age, gender, BMI, hypertension, and diabetes mellitus.

## Appendix A

**Table A1:** Biomarker concentrations by estimated glomerular filtration rate (eGFR).

	**eGFR <30 mL/min/1.73 m²**		**eGFR 30–44 mL/min/1.73 m²**		**eGFR 45–59 mL/min/1.73 m²**		**eGFR 60–89 mL/min/1.73 m²**	
**Biomarker**	**median**	**IQR**	**median**	**IQR**	**median**	**IQR**	**median**	**IQR**
sST2 (pg/mL)	3998	3262–7949	3612	2997–5689	4167	2643–6620	3731	2660–6098
GDF-15 (pg/mL)	1816	1420–2139	1086	883.4–1567	843.8	602.5–1064	929.7	683.0–1084
H-FABP (ng/mL)	4.4	3.5–6.2	2.9	2.1–3.7	2.2	1.8–3.0	1.4	1.0–1.9
IGF-BP2 (ng/mL)	177.8	127.5–309.0	135.4	94.9–198.8	126.0	91.3–182.5	122.6	79.0–171.4
suPAR (pg/mL)	3342	2618–3977	2443	1936–2921	1898	1537–2382	1811	1422–2442
**eGFR ≥ 90 mL/min/1.73 m²**		**total cohort**						
**median**	**IQR**	**median**	**IQR**	***p*-value**				
5170	2820–11,952	3870	2898–6641	0.788				
506.3	348.3–896.4	975.4	745.5–1316	<0.0001				
1.0	0.8–1.6	2.4	1.6–3.4	<0.0001				
59.9	50.2–83.6	127	87.7–188.1	0.001				
1648	1364–2393	2153	1694–2801	<0.0001				

**Table A2:** Multiple linear regression analysis with adjustment for age, gender, BMI, hypertension and diabetes mellitus.

Dependent Variable: eGFR					Dependent Variable: UACR				
Adjustment for: Age, Gender, BMI, Hypertension, Diabetes					Adjustment for: Age, Gender, BMI, Hypertension, Diabetes				
Biomarker	r	Std. Error	95% CI	*p*-Value	Biomarker	r	Std. Error	95% CI	*p*-Value
sST2 (pg/mL)	0.000	0.000	0.000–0.001	0.643	sST2 (pg/mL)	0.031	0.011	0.008–0.053	0.007
GDF-15 (pg/mL)	−0.010	0.002	−0.013–(−0.007)	<0.0001	GDF-15 (pg/mL)	0.179	0.071	0.040–0.319	0.012
H-FABP (ng/mL)	−1.187	0.321	−1.820–(−0.555)	<0.0001	H-FABP (ng/mL)	17.542	12.923	−7.938–43.02	0.176
IGF-BP2 (ng/mL)	−0.064	0.012	−0.087–(−0.041)	<0.0001	IGF-BP2 (ng/mL)	2.086	0.464	1.170–3.001	<0.0001
suPAR (pg/mL)	−0.006	0.001	−0.008–(−0.004)	<0.0001	suPAR (pg/mL)	0.084	0.045	−0.004–0.171	0.062

Variance inflation factor (VIF): age = 1.037, gender = 1.084, BMI = 1.197, hypertension = 1.122, diabetes mellitus = 1.229. Abbreviations: eGFR = estimated glomerular filtration rate, UACR = urinary albumin/creatinine ratio, B = regression coefficient, BMI = body mass index, eGFR = estimated glomerular filtration rate, 95% CI = 95% confidence interval.
