# Supplementary material for: Novel Biomarkers in Patients with Chronic Kidney Disease: An Analysis of Patients Enrolled in the GCKD-Study
Source: J Clin Med. 2020 Mar 24;9(3):886. doi: 10.3390/jcm9030886 (PMC7141541; doi:10.3390/jcm9030886)
Supplement: Supplementary file 1 [file jcm-09-00886-s001.pdf]

Supplementary Materials: Novel Biomarkers in Patients with Chronic Kidney Disease: An Analysis of Patients Enrolled in the GCKD-Study

Moritz Mirna [1], Albert Topf [1], Bernhard Wernly [1], Richard Rezar [1], Vera Paar [1], Christian Jung [2], Hermann Salmhofer [3], Kristen Kopp [1], Uta C. Hoppe [1], P. Christian Schulze [4], Daniel Kretzschmar [4], Markus P. Schneider [5], Ulla T. Schultheiss [6], Claudia Sommerer [7], Katharina Paul [8], Gunter Wolf [8], Michael Lichtenauer [1] and Martin Busch [8]

Table A1. Biomarker concentrations by estimated glomerular filtration rate (eGFR).

	eGFR <30 mL/min/1.73 m²		eGFR 30–44 mL/min/1.73 m²		eGFR 45–59 mL/min/1.73 m²		eGFR 60–89 mL/min/1.73 m²		eGFR ≥ 90 mL/min/1.73 m²		total cohort		
**Biomarker**	**median**	**IQR**	**median**	**IQR**	**median**	**IQR**	**median**	**IQR**	**median**	**IQR**	**median**	**IQR**	**p-value**
sST2 (pg/mL)	3998	3262–7949	3612	2997–5689	4167	2643–6620	3731	2660–6098	5170	2820–11,952	3870	2898–6641	0.788
GDF-15 (pg/mL)	1816	1420–2139	1086	883.4–1567	843.8	602.5–1064	929.7	683.0–1084	506.3	348.3–896.4	975.4	745.5–1316	<0.0001
H-FABP (ng/mL)	4.4	3.5–6.2	2.9	2.1–3.7	2.2	1.8–3.0	1.4	1.0–1.9	1.0	0.8–1.6	2.4	1.6–3.4	<0.0001
IGF-BP2 (ng/mL)	177.8	127.5–309.0	135.4	94.9–198.8	126.0	91.3–182.5	122.6	79.0–171.4	59.9	50.2–83.6	127	87.7–188.1	0.001
suPAR (pg/mL)	3342	2618–3977	2443	1936–2921	1898	1537–2382	1811	1422–2442	1648	1364–2393	2153	1694–2801	<0.0001

Table A2. Multiple linear regression analysis with adjustment for age, gender, BMI, hypertension and diabetes mellitus.

**dependent variable: eGFR** adjustment for: age, gender, BMI, hypertension, diabetes					**dependent variable: UACR** adjustment for: age, gender, BMI, hypertension, diabetes				
Biomarker	r	Std. error	95% CI	p-value	Biomarker	r	Std. error	95% CI	p-value
sST2 (pg/mL)	0.000	0.000	0.000–0.001	0.643	sST2 (pg/mL)	0.031	0.011	0.008–0.053	0.007
GDF-15 (pg/mL)	−0.010	0.002	−0.013–(−0.007)	<0.0001	GDF-15 (pg/mL)	0.179	0.071	0.040–0.319	0.012
H-FABP (ng/mL)	−1.187	0.321	−1.820–(−0.555)	<0.0001	H-FABP (ng/mL)	17.542	12.923	−7.938–43.02	0.176
IGF-BP2 (ng/mL)	−0.064	0.012	−0.087–(−0.041)	<0.0001	IGF-BP2 (ng/mL)	2.086	0.464	1.170–3.001	<0.0001
suPAR (pg/mL)	−0.006	0.001	−0.008–(−0.004)	<0.0001	suPAR (pg/mL)	0.084	0.045	−0.004–0.171	0.062

Variance inflation factor (VIF): age = 1.037, gender = 1.084, BMI = 1.197, hypertension = 1.122, diabetes mellitus = 1.229. Abbreviations: eGFR = estimated glomerular filtration rate, UACR = urinary albumin/creatinine ratio, B = regression coefficient, BMI = body mass index, eGFR = estimated glomerular filtration rate, 95% CI = 95% confidence interval.
